# Contactless Breathing Monitoring at Home and in the Hospital: Protocol for a Low-Cost Frequency-Modulated Continuous-Wave Radar-Based Device

**DOI:** 10.2196/59532

**Published:** 2025-02-25

**Authors:** Arnav Hari, Ravishankar Kumar, Brijesh Kumbhani, Sam Darshi, Satyam Agarwal, Jyotindra Singh Sahambi, Suksham Jain, Deepak Chawla

**Affiliations:** 1 Department of Electrical Engineering Indian Institute of Technology Ropar Rupnagar India; 2 Government Medical College & Hospital Chandigarh India

**Keywords:** radar, contactless vital monitoring, breathing rate monitoring, vital signs, health monitoring

## Abstract

**Background:**

Contactless monitoring of vital signs, especially the breathing of children, in the hospital is performed on a priority basis because their organs and immune system are immature. Therefore, continuous monitoring of their vital signs with a sensor that is directly attached to their body is not possible, as it irritates the sensitive newborn skin and causes discomfort. A contactless frequency-modulated continuous-wave (FMCW) radar-based device can wirelessly monitor the breathing rate and pattern of a child in the hospital or at home. Signal-processing capability can be added to this device to process breathing data and analyze the apnea condition arising due to irregular breathing patterns.

**Objective:**

This study will develop a contactless FMCW radar-based system to accurately monitor the breathing rate and pattern of neonates and infants in hospitals and at home in order to provide a noninvasive, nonintrusive and contactless alternative to conventional sensor-based methods and address a critical need in neonatal care, potentially improving health outcomes for vulnerable infants.

**Methods:**

The radar transmits a signal toward the body, and the time taken by the signal received to travel from the body to the receiving antenna is analyzed. This time is proportional to the distance between the radar and the body, and the breathing pattern is recognized as a slight, periodic variation in this distance. We will use this concept with multiple antenna systems to monitor the breathing of neonates with improved sensitivity. The radar-based device will be installed, in addition to conventional breathing monitors, in the neonatal intensive care unit. The signals received at the radar and the respiration signals from conventional monitors will be recorded in a database. Signal-processing techniques will be applied to extract breathing signals from the signals received at the radar.

**Results:**

This study was funded in January 2023 by the Science and Engineering Research Board (SERB) of India. The device was designed by May 2024, and a working proof-of concept was verified in the Indian Institute of Technology (IIT) Ropar laboratory. Implementation of the proposed method for initial study began in December 2024. Results are expected to be published in the first quarter of 2025.

**Conclusions:**

The contactless FMCW radar-based system will provide reliable estimation of the breathing rate and pattern, which is close to the conventional reference device values most of the time. Our device will also provide a seamless breathing-monitoring system to be used both in hospitals and at home for newborns and premature babies until they are fully healthy and fit.

**International Registered Report Identifier (IRRID):**

PRR1-10.2196/59532

## Introduction

In recent times, we have seen advancements in the medical sector, especially in health monitoring, by replacing conventional medical equipment with wireless equipment, which is convenient and accurate for usage. Such advancements have made some vital sign monitoring possible at the home of the patient/subject. As an advanced health-monitoring system, smart homes monitor not only temperature and the environment but also some critical vital signs [1]. Continuously monitoring the breathing rate benefits patient management by early detection of health deterioration and breathing irregularities, if any. For monitoring children and newborns, it is convenient if performed at home. Nearly 15 million infants born prematurely in the world every year need to be monitored until they are healthy and fit [2]. In these children, basic vital parameters, such as breathing, the heart rate, and oxygen saturation, are especially monitored. Initially, these parameters are monitored by a sensor that is directly attached to their body [3], which, however, causes skin irritation and may also lead to pressure necrosis. Regularly observing a patient’s vital signs aids medical professionals in identifying early abnormalities and assessing the progression of an illness, as well as evaluating the effectiveness of a treatment method. Contactless monitoring of a patient’s vital signs, minimizes inhibition, reduces the risk of infection, and eliminates any discomfort to their skin [4]. Radar is already a promising technology for wireless health parameter monitoring due to its low power, low cost, and security purposes. In this paper, an FMCW radar-based device [5] is proposed to investigate breathing patterns in infants in different scenarios. The device is intended to be used in the hospital or at home for monitoring purposes. Using the proposed design, we will eliminate the requirement of a cannula, which is usually attached to the patient’s nose for flow-based breathing-monitoring purposes. Another method that is used as an alternative to interpolate normal breathing is a pulse oximeter, which is attached to the patient’s finger for oxygen saturation measurement that correlates with normal/abnormal breathing. Such methods come with the requirement of one or the other kind of attachment to the patient’s body, which might create irritation or discomfort for the patient. Thus, we will use a contactless monitoring device for breathing pattern/rate measurement. In this paper, the feasibility of radar technology as a possible means of continuously monitoring vital signs without physical contact will be studied. Radar means detecting an object and estimating its parameters based on its distance from the radar. Continuous-wave radar is used in the microwave range of the spectrum, which means transmit frequencies are within tens of gigahertz [6]. The contactless FMCW radar-based model is based on wireless technology, which includes radio frequency for the transmission of signals and reception after reflection so that direct contact with the patient is not needed, providing an advantage over conventional methods by monitoring health parameters remotely, which is suitable in emergencies and allows health care providers to assess the patient’s condition without being physically present at that place. In this study, we propose a device for contactless breathing rate monitoring. In particular, this device is targeted to be used for the contactless breathing monitoring of newborns to reduce discomfort and potential infection risks due to wired sensor devices by implementing a radar system that will be kept at some distance from the babies so that they do not face any problem during the monitoring of vital signs, especially the respiration rate. For this purpose, we will use the radar for measuring inhalation and exhalation through the outward or inward movement of the abdominal area. This will be monitored in the form of changes in the distance from the radar and the patient being monitored.

## Methods

### Study Setting

The proposed study will be jointly performed at the Indian Institute of Technology (IIT) Ropar and Government Medical College and Hospital (GMCH) Chandigarh. IIT Ropar is a technology education institute having ~2500 students [7]. The campus also accommodates the staff and faculty members in the on-campus residential facility. The device will be developed at IIT Ropar. The initial study will be conducted on healthy students of the institute who volunteer to be part of the study. The accuracy of the breathing pattern of a healthy human being will be verified. Next, healthy children of the staff and faculty members residing on campus will be enrolled after obtaining informed consent from their parents. Furthermore, a study will be conducted at GMCH Hospital’s pediatrics department, which has an inpatient facility for all age groups, including newborn and premature neonates.

### Study Design

**Figure 1:**
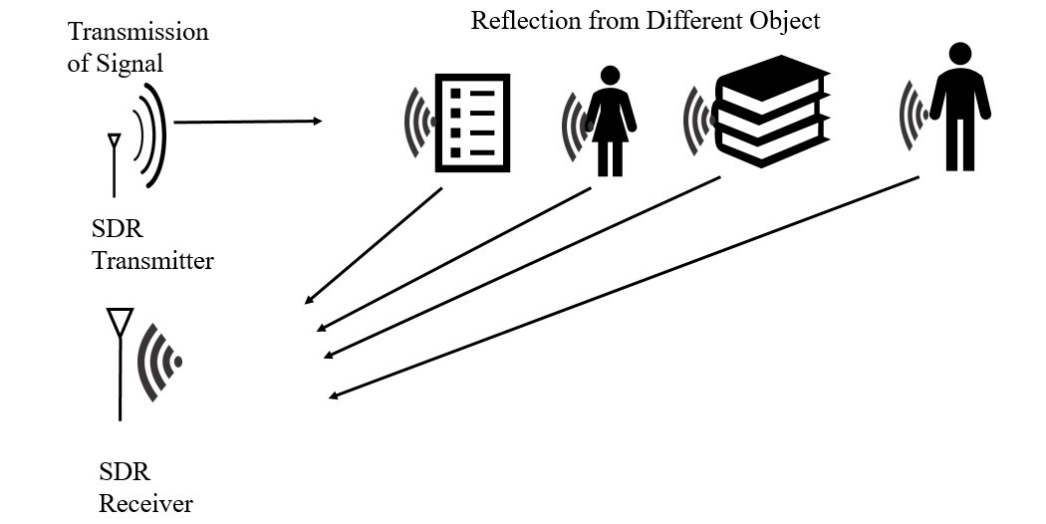
Separating different objects based on reflection. SDR: software-defined radio.

The proposed study is intended to prove the applicability of contactless methods for breathing rate monitoring in neonates. Further, this study will be useful in the detection and management of apnea conditions due to irregularity in breathing patterns. The primary objectives of the study are as follows:

- Designing a device for noncontact breathing rate monitoring using the FMCW radar principle, which transmits a signal with the frequency varying linearly with time. When this signal reflects off an infant’s chest, the received signal has a frequency difference (beat frequency) from the transmitted signal. This frequency difference is proportional to the minute movements caused by breathing. Thus, the beat frequency carries information of the breathing pattern. In addition to the breathing pattern, some unwanted reflected components from multiple objects around the patient being monitored are contained in the radar signal, as illustrated in Figure 1. The primary objective is to design algorithms to suppress the effect of reflections from stationary objects.
- Identifying specific signal fluctuations related to the breathing of the patient being monitored and distinguishing these variations from other persons in the room or other body movements, such as those of limbs. Breathing is periodic, so the beat frequency variation is also periodic. Variation due to limb movements and other people is large and aperiodic. Therefore, signal processing techniques, including filtering, can be used to minimize the effects of unwanted reflections and movements.
- Further extending this setup to analyze breathing signals of multiple persons and separating them.

### Prototype and Implementation

**Figure 2:**
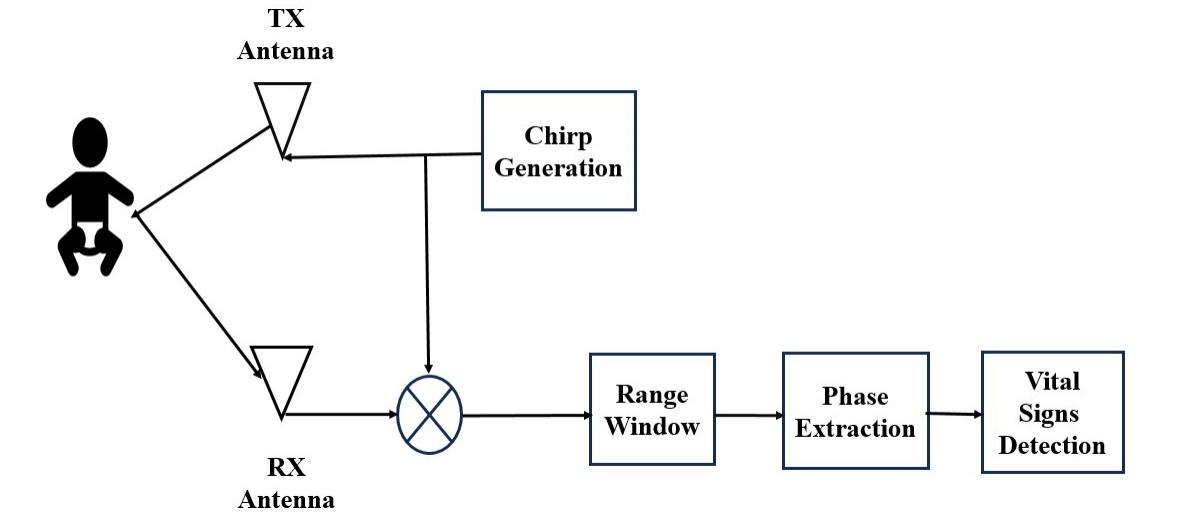
System model design. RX: receiving; TX: transmission.

The main objective of the model is to monitor the breathing of premature and newborn babies without any contact with their bodies. The proposed monitoring device is contactless. Therefore, it will not cause skin injury, irritation, allergy, or infection. Device accuracy is targeted to be more than 90% in all scenarios and more than 98% in a controlled setup [1]. The controlled setup is defined as a laboratory environment or the target patient being instructed to restrict any movement of body parts. This model combines the development of hardware and software, in addition to the testing protocol for the breathing rate. The software contains signal-processing algorithms to clean the received signal after reflection from the target and the other objects or persons in the vicinity, as shown in Figure 2. The waveform is designed and generated to be sent toward the patient being monitored using a software-defined radio (SDR) transmitter [8,9]. The same SDR is used to receive the reflected signal. The broadcast signal is reflected and received with a time delay due to the distance propagated between the device and the target patient. The difference in frequency between the transmitted and received signals, which determines the beat frequency, provides distance information. The distance varies with the state of breathing, such as inhalation results in reduced distance and exhalation results in increased distance due to expansion and contraction of the chest and abdominal region of the patient. The reflected signal’s components are reflected from the patient and nearby objects. The reflected signal received at the SDR receiver is converted back to the baseband after dechirping. The dechirped signal is processed further in a general-purpose computer to extract the required breathing signal by mitigating the effects of the reflections from objects other than the patient being monitored. This requires passing the signal through a low-pass filter to obtain the frequency of interest (ie, to retain frequency components that are within the range of the breathing rate). We will also implement the algorithms to separate the reflection from different objects based on their reflection time so that the objects at different distances are separated. Wireless signals travel at the speed of light. Separating these reflections eliminates them from static objects, as their reflections do not change over time [8].

### FMCW Radar and Study Measurement

Effective contactless monitoring of each baby in the hospital was initiated after stability criteria were met, such as respiratory status without serious illness. FMCW radar monitoring is performed continuously during the monitoring period [10]. The benefit of this device is that it can be used in hospitals and homes both. Many times, it is only necessary to monitor the breathing, which can be performed at home with the proposed device without admitting the child to the hospital. For the proposed study and initial trial, the infants will be observed with their chest facing the radar at a distance of roughly 1-1.5 m from the low-vibration tripod to which the radar is attached. A research assistant will track each infant’s vital signs for 10 minutes, 3 times a day. The effectiveness of the radar-based breathing rate–monitoring device will be evaluated by comparing the device’s measurements with clinical recordings.

### Sample Size and Data Analysis

A sample of 20-30 premature newborn babies will be included in the study. Data collection will be performed with the help of the neonatology department at GMCH Chandigarh. The radar-based device will be installed, in addition to conventional breathing monitors, in the neonatal intensive care unit. The signals received at the radar and the respiration signals from the conventional monitors will be recorded in a database. An in-depth analysis of radar signals received from each infant will be carried out. All the factors critical for understanding the complexities of the premature newborn condition and care will be observed, providing the diversity and depth of experience necessary for such research [11]. The main idea is to investigate different effects of collecting radar data from the chest and abdominal region, removing the effect of any interference or movements and reflections from the surroundings. Further analysis and algorithm development will be carried out to extract breathing signals when each infant is with the mother. If monitoring of twins is possible, a minimum safe distance will be required to mitigate the radar interface between them. The basic principle that will guide the data collection protocol will ensure seamless operation at GMCH Chandigarh.

### Ethical Considerations

The study was approved by GMCH Chandigarh and the Government of India. Informed consent will be obtained from parents. They will be informed about the purpose of the experiment, the procedure, and benefits. We will ensure that they do not face any type of harm and that they voluntarily agree to participate. Data confidentiality will be ensured, and participants’ identities will be protected. Ethical approval (approval number GMCH/IEC/2022/730) was obtained from the GMCH Chandigarh Institutional Review Board (IRB) or Ethics Committee to ensure that the study meets ethical standards and that participants are protected from potential harm.

## Results

Data collection and implementation of the proposed device was performed with the help of IIT Ropar and GMCH Chandigarh. The study was funded in January 2023 by the Science and Engineering Research Board (SERB) of India. The device was designed by May 2024, and a working proof-of concept was verified in the IIT Ropar laboratory. Implementation started in December 2024. Initially, we will apply this method to monitor a single child, and then, this study will be extended to monitor multiple infants by modifying the device design and signal-processing algorithms. The main outcome of the proposed method is to obtain breathing patterns of infants at home and in hospitals to predict their health condition based on the breathing patterns. We expect to obtain the results of monitoring a single child by the end of the first quarter of 2025.

## Discussion

### Summary

The proposed study will develop a device for accurate noncontact monitoring of the breathing rate and patterns of neonates and infants in the hospital and at home using an FMCW radar-based system. The working principle of the device is based on capturing, using radar, the pattern of movements in the chest and abdominal region due to inhalation and exhalation. This will address the critical need for noninvasive and noncontact continuous respiration monitoring in clinical settings. With the system being noncontact and portable, it can also be used at home to monitor the patient’s condition. Unlike wearable devices that may be affected by environmental factors and require direct contact, the proposed system offers a significant advantage over conventional chest-mounted devices that may cause discomfort and privacy concerns. The typical range for an infant’s respiratory rate is between 40 and 60 breaths per minute [12]. A breathing rate outside this range indicates that the patient is not healthy. Through the usage of the proposed technology, we plan to demonstrate high accuracy in identifying respiratory irregularities, such as apnea, aligning with the study’s objective to enhance neonatal care. Contactless radar systems [13-17] have been proven to be a more convenient option for users compared to wearable devices due to immunity to environmental constraints, wider coverage, the absence of privacy concerns, and importance for heavy-burned and newborn babies, enhancing usability. The ability to detect vital signs, particularly the breathing rate, is typically required to identify human presence in short-range environments. However, the system’s accuracy may be affected by movement artifacts, physical abstraction, clothing, cobedding, and positioning. Other constraints related to the proposed method are a trade-off between range and resolution due to bandwidth constraints, and further validation in diverse clinical settings is required. Despite these limitations, the signal-processing techniques will be used to improve the performance and bring about significant advancements in neonatal respiratory care, with broader implications for improving patient care and monitoring in various clinical and home settings. Once the designed device is developed, it will be tested in the neonatology department of GMCH Chandigarh, where breathing rate monitoring will be compared with conventional methods. We will have a database of signals received at the proposed device and those from conventional devices. This database can be used to test various algorithms. Future research will focus on refining the device’s accuracy and expanding its applicability to a wider range of patients and conditions. The device design can be modulated to monitor multiple individuals through use of multiple antennas and advanced signal-processing techniques.

### Conclusion

An FMCW device can measure submillimeter-range shifts because of its improved precision in measuring displacements. Breathing using an FMCW radar-based device [18-20] is useful for pathologies that are difficult to detect solely with the use of an electrocardiogram (ECG). FMCW devices are cheap and easy to operate. Our model will also have the advantage of better resolution compared to other radar-based model designs and will be based on radio frequency signals from a radar to detect the patient’s position and extract respiration data [21-23]. With the help of this system, we can detect the breathing of patients within the range of up to 45 cm and possibly improve spatial resolution and range with additional radar and antenna units.

## Abbreviations

FMCW: frequency-modulated continuous-wave
GMCH: Government Medical College and Hospital.
IIT: Indian Institute of Technology
SDR: software-defined radio.

## Data Availability

The data from this trial will be accessible to the public and research community. The research team will decide on the dissemination method once the trial ends and our results are published. Although the data will not be made immediately public, they can be requested from the corresponding author.
